# Endothelium-dependent vasorelaxant effects of praeruptorin a in isolated rat thoracic aorta

**DOI:** 10.1080/21655979.2022.2062979

**Published:** 2022-04-13

**Authors:** Zhenkun Li, Fengrong Zhang, Shicong Wang, Honghe Xiao, Jingyi Wang, Xianyu Li, Hongjun Yang

**Affiliations:** a Beijing Key Laboratory of Traditional Chinese Medicine Basic Research on Prevention and Treatment for Major Diseases, Experimental Research Center, China Academy of Chinese Medical Sciences, Beijing, China; b Beijing Big Brand League Technology Consulting Co., Ltd, Beijing, China; c Institute of Chinese Materia Medica, China Academy of Chinese Medical Sciences, Beijing, China

**Keywords:** Praeruptorin A, vasodilative effect, mechanism, thoracic aorta artery rings

## Abstract

Praeruptorin A (PA) is a natural coumarin compound from the roots of Radix Peucedani and is commonly used in the treatment of certain respiratory diseases and hypertension. Although previous studies identified relaxant effects of PA on tracheal and arterial preparations, little is known about its vasodilative effects and underlying mechanisms. Here, an organ bath system and tension recording methods were used to prepare and analyze isolated rat thoracic aorta artery rings. Aorta artery rings were pre-contracted with phenylephrine and then incubated with PA, and the possible mechanism of relaxation was investigated by adding inhibitors of nitric oxide synthase (NG-nitro-L-arginine methyl ester, L-NAME), endothelial nitric oxide synthase (L-NG-nitroarginine, L-NNA), cyclooxygenase (indomethacin), guanylyl cyclase (1 H-[1,2,4]oxadiazolo [4,3-a]quinoxalin-1-one, ODQ), and KCa channels (tetraethylammonium, TEA). Our study showed that PA-induced vasodilation was blocked by L-NAME, L-NNA, and ODQ, while CaCl_2_-induced vasoconstriction was countered by PA. Thus, PA may exert a vasodilatory effect by influencing the amounts of endothelium-derived relaxing factors through endothelial-dependent NO-cGMP and prostacyclin pathways (such as NO and prostacyclin 2). In the rat thoracic aorta, PA reduces vasoconstriction by inhibiting Ca^2+^ inflow.

## Graphical abstract

**Figure:**
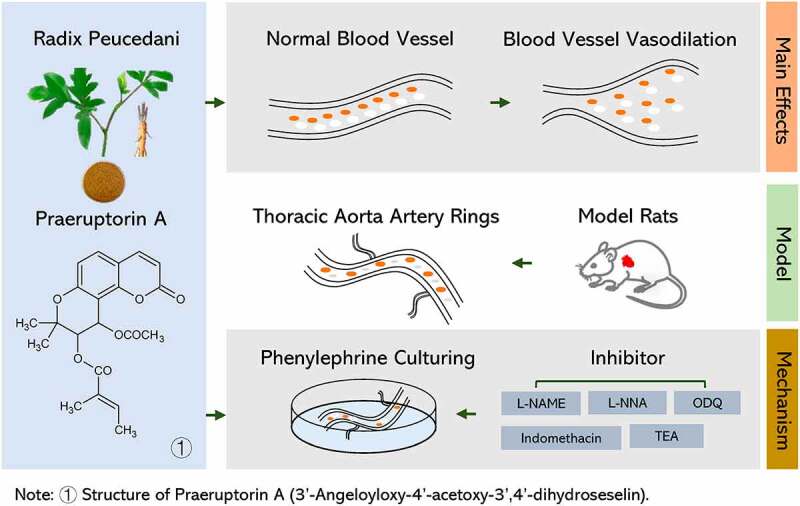

## Introduction

Radix Peucedani (in Chinese, Qian Hu), the dried roots of *Peucedanum praeruptorum* Dunn., were first described in *Miscellaneous Records of Famous Physicians* (in Chinese, *Ming Yi Bie Lu*). It has a long history of use in diseases of the lung and respiratory system, such as cough, dyspnea, and upper airway infections [1]. Qian Hu consists of a great number of chemical components, but only a few of these components have been revealed as the main bioactive constituents. These bioactive components include angular-type pyranocoumarins, (+)-praeruptorin A, (±)-praeruptorin B, (+)-praeruptorin B and (+)-praeruptorin E. These compounds have various pharmacological activities, involving vasodilation, neuroprotection, cardioprotection, hepatoprotection, and anti-platelet aggregation effects [2–4]. One of these compounds in particular, praeruptorin A (PA), has been recognized as the main active component of Qian Hu, and it has been shown to have anticancer, antihypertension, and anti-inflammatory properties [5–9].

Endothelial-dependent vasodilation can be decreased gradually by chronic hypertension, diabetes, and cardiovascular disease and by other factors [10]. The chemical entities that mediate endothelium-dependent vasodilation may be divided into calcium antagonists, alpha 1-adrenergic antagonists, cAMP-elevating drugs, and cGMP-elevating agents. It is generally accepted that calcium-dependent phosphorylation of myosin light chains initiates smooth muscle contraction. A variety of evidence suggests that certain vasodilators inhibit calcium-dependent phosphorylation of myosin light chains and smooth muscle contraction via activation of cAMP and cGMP-dependent protein phosphorylation [11].

Analogues of L-arginine are widely used inhibitors of nitric oxide synthase (NOS) activity both *in vitro* and *in vivo*, with NG-nitro-L-arginine methyl ester (L-NAME) being a commonly used analogue [12]. Acute and chronic L-NAME treatment leads to changes in blood pressure and vascular reactivity due to decreased nitric oxide (NO) bioavailability [13]. NO is of major importance in the control of systemic vessels, but not coronary resistance vessels. At rest and during exercise, and after treatment with L-NG-nitroarginine (L-NNA), the cyclooxygenase pathway has been found to be involved in myocardial reactive hyperemia and in the residual relaxation response to bradykinin of isolated coronary arteries [14].

Importantly, NO has both inhibitory and excitatory effects on all types of respiratory neurons. Inhibitory effects involve soluble guanylyl cyclase, and they are blocked with 1 H-[1,2,4] oxadiazolo [4,3-a] quinoxalin-1-one (ODQ), whereas excitation is antagonized by uric acid and possibly mediated via peroxynitrite [15]. In addition, indomethacin may have more potent vasoconstrictive activity and unique direct neuronal or NO-dependent inhibitory pathway activity [16]. In blood vessels, NO causes hyperpolarization, suggesting that ATP-dependent potassium channels are involved the entire vasorelaxation process. Other calcium-dependent potassium channels (KCa) that are inhibited by small concentrations of tetraethylammonium (TEA) are likely to be involved in this same general vasorelaxant effect. Hence, we predict that multiple compounds with inhibitory activity, such as L-NAME (a nitric oxide synthase inhibitor), L-NNA (an endothelial nitric oxide synthase inhibitor), indomethacin (a cyclooxygenase inhibitor), ODQ (a guanylyl cyclase inhibitor, and TEA (a KCa channel inhibitor, might have substantive impacts on vasorelaxation.

Although it is known that PA induces tracheal smooth muscle relaxation after pre-constriction with KCl and acetylcholine [3] and may open K_ATP_ channels [17], the mechanisms behind these effects remain obscure. Therefore, in this study we aimed to explore the possible factors involved in the vasorelaxant effect of PA with a rat thoracic aorta ring model and by investigating interactions with important endothelium-dependent vasodilators.

## Materials and methods

### Animals

Adult male Sprague-Dawley rats weighing 260 ± 10 g were obtained from the Animal Breeding Center of Beijing Vital River Laboratories Company (Beijing, China). All rats were housed at 22 ± 2°C with a relative humidity of 50 ± 10% and a 12 h light/12 h dark cycle. The animals had free access to water and food. All animal experiments were approved by the Ethics Committee of the China Academy of Chinese Medical Sciences (No.20162002) and were performed according to the *Guide for the Care and Use of Laboratory Animals* (Council, 2010).

### Drugs

The drugs used in this study were: PA (PHL83553, Sigma, USA), indomethacin (PHR1247, Sigma, USA), L-phenylephrine chloride (PE) (PHR1017, Sigma, USA), acetylcholine chloride (A2662, Sigma, USA), L-NAME (N5752, Sigma, USA), L-NG-nitroarginine (L-NNA) (N5501, Sigma, USA), 1 H-[1,2,4]oxadiazolo[4,3-a]quinoxalin-1-one (ODQ) (495,320, Sigma, USA). Indomethacin was dissolved in 0.1 M Na_2_CO_3_ solution. ODQ and PA were prepared in 20% dimethyl sulfoxide (DMSO). Others were dissolved in distilled water. The final concentration of DMSO in the bath was less than 0.5%, which had no influence on vasodilatation [12].

### Preparation of isolated rat thoracic aortas

An organ bath system (ALC-M, Shanghai, China), an electronic analytical balance (BP110S, Sartorius, Germany), and a tension recording method were used to prepare isolated rat thoracic aorta artery rings. The thoracic aorta was dissociated, and then connective and fat tissue were removed in a 4°C K-H solution (118.96 M NaCl, 4.73 mM KCl, 1.17 mM KH_2_PO_4_, 1.17 mM MgSO_4_, 25.0 M NaHCO_3_, 1.35 M CaCl_2_, and 11.1 M glucose, pH 7.4). Aorta artery rings (2 mm) were suspended by cotton threads in an organ bath containing 5 mL K-H solution, maintained at 37 ± 0.1°C and gassed with 95% O_2_ + 5% CO_2_. Rings were first stabilized with a 1.0 g weight to establish a tension of 9.8 mN for 1 h, and the solution was changed every 20 min during this period [18]. The isometric contraction was recorded by a force transducer (MPA 2000, Shanghai, China) coupled to an amplifier-recorder and also to a personal computer equipped with an analog-to-digital converter board. In endothelium removal experiments, the endothelial layer was removed by gently rubbing the intimal surfaces of vessels with a cotton ball. The presence of functional endothelium was assessed by applying acetylcholine (1 μM), which induced more than 70% relaxation of vessels after precontraction with 1 μM PE. Less than 10% of relaxation caused by acetylcholine after PE pretreatment was considered to indicate endothelium denudation [19,20].

### Effects of PA on vasorelaxation in endothelium-denuded vessel rings

PE (1 μM) was employed as the contractile agent. After stable contraction was established, constant volumes of increasing concentrations of PA (2.77 × 10^−7^ to 2.04 × 10^−4^ M) were cumulatively applied every 10 min. Vasodilatation of the endothelium-denuded vessels caused by PA was analyzed by comparing responses to those of intact vessel rings derived from the same aorta.

### Endothelium-derived relaxing factor (EDRF) inhibitors in PA-induced vasorelaxation

Sustained contraction was induced in intact aorta rings by the addition of PE (1 µM) after 15 min incubation with L-NAME (300 μM), an endothelial nitric oxide synthase inhibitor (L-NNA, 300 μM), or a nonselective inhibitor of cyclooxygenase (COX) (indomethacin, 10 μM). PA (2.77 × 10^−7^ to 2.04 × 10^−4^ M) was then cumulatively applied after stable contraction was established. Thus, concentration-response curves to PA (2.77 × 10^−7^ to 2.04 × 10^−4^ M) were obtained. Control experiments were performed without pre-incubation with L-NAME, L-NAA, or indomethacin.

### Effect of guanylyl cyclase inhibition on PA-induced vasorelaxation

Sustained contraction was induced by PE (1 µM) in intact aorta rings that were previously incubated for 15 min with ODQ (10 μM), a soluble guanylyl cyclase inhibitor [21]. Then, concentration-response curves to PA (2.77 × 10^−7^ to 2.04 × 10^−4^ M) were obtained according to the method described above. The same experiment protocol was applied to the control group, except for incubation with ODQ [22].

### Effect of KCa channel inhibition on PA-induced vasorelaxation

Sustained contraction after PE (1 µM) incubation was obtained in intact aorta rings that were pretreated with tetraethylammonium (TEA, 10 mM), a KCa channel blocker, for 15 min, and concentration-response curves to PA (2.77 × 10^−7^ to 2.04 × 10^−4^ M) were subsequently obtained based on the methods described above. The same experimental protocol without incubation with TEA was used for the control group.

### Role of calcium in PA-induced vasorelaxation

Denuded and intact rings were incubated in K-H solution without calcium for 1 h [23]. During this period, the solution was changed every 20 min. Aorta rings were first incubated with PA (2.76 × 10^−^[5] M) for 10 min, and then CaCl_2_ (6.00 × 10^−5^ to 4.55 × 10^−3^ M) was cumulatively applied every 5 min after contractile responses were induced by 1 μM PE and 60 mM KCl. Dose-response curves of aorta rings were recorded. The control group was conducted with the same experiment protocol in the absence of PA.

### Data analysis

All data are presented as means ± SD. Relaxation rate was expressed as a percentage of the vasodilative response induced by PA to vasoconstriction caused by various contractile agents. The maximal relaxation response of PA was expressed as a percentage (E_max_) and as an EC_50_, which is the concentration of the test compound that causes 50% of its maximal response (The equation of EC50 from GraphPad Prism: Y = Bottom+ (Top-Bottom)/(1 + 10^((LogEC50-X)*Hill Slope))). Values of responses to PA were calculated by GraphPad Prism software. Relaxation potency was expressed as the negative logarithm of the EC_50_ (pEC_50_). Two-way repeated measured ANOVA followed by Bonferroni’s multiple comparison was used for data analysis [24]. Differences for which *p*< 0.05 were considered statistically significant.

## Results

### Experimental workflow

**Figure 1.**
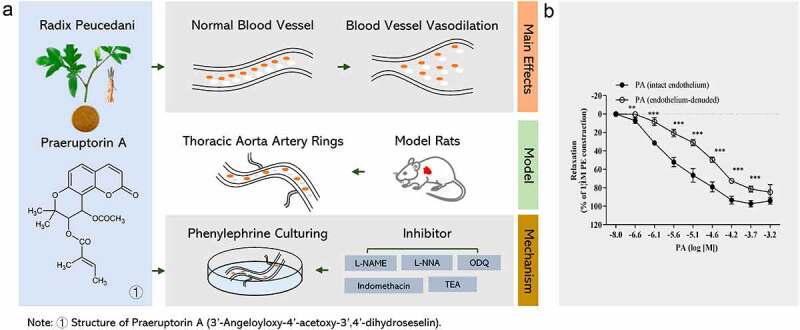
(A) Schematic diagram of experimental procedures. (B) Effect of PA on tension in PE (1 μmol/L) pre-contracted aortic rings with endothelium (+E, n = 5) or without endothelium (-E, n = 5). Relaxation (%) indicates the percentage of PE-induced contraction. Values are expressed as the mean ± SD. *p < 0.05, **p< 0.01, ***p< 0.001 vs the endothelium-denuded rings group.

Figure 1Aprovides a schematic representation of our experimental approach. To explore the relaxant effect of PA from the spectrum of the endothelium, we followed this workflow to profile the differential protein expression in isolated rat thoracic aorta rings. The aorta artery rings were then pre-contracted, and PE was added. After that, the pre-contractive model was incubated with PA, and possible mechanisms of action were investigated by adding inhibitors to various proteins, including EDRF (L-NAME), endothelial nitric oxide synthase (L-NNA), COX (indomethacin), guanylyl cyclase (ODQ), and KCa channels (TEA). Ultimately, these studies were designed to reveal the mechanism by which PA induces endothelium-dependent vasorelaxation and smooth muscle relaxation (Table 1).

**Table 1.** Roles of different treatments on vasorelaxant effects of PA after PE-induced contractions in intact thoracic aorta rings

Treatments	n		pEC_50_ ± SD	E_max_ ± SD (%)	Two-way ANOVA analysis
control Removed Endothelium-denuded		5 5	5.63 ± 0.15 4.83 ± 0.14***	98.06 ± 4.33 91.52 ± 8.07	*p* < 0.001
control +300 µM L-NAME	4 4		4.86 ± 0.09 4.57 ± 0.07**	97.13 ± 3.70 94.13 ± 7.15	*p*< 0.01
control +10 µM Indomethacin	4 4		4.99 ± 0.28 4.58 ± 0.11*	95.55 ± 6.65 91.05 ± 7.65	*p*< 0.05
control +100 µM L-NNA	4 4		5.02 ± 0.25 4.90 ± 0.29	97.5 ± 13.10 95.16 ± 7.17	*p* > 0.05
control +10 µM ODQ	4 4		4.89 ± 0.16 4.40 ± 0.10**	90.97 ± 1.70 89.16 ± 7.54	*p* < 0.01
control +10 µM TEA	4 4		5.27 ± 0.22 5.38 ± 0.15	110.9 ± 5.46 107.06 ± 5.12	*p* < 0.05

Compared with control: *p< 0.05, **p < 0.01, ***p< 0.001;

### In phenylephrine (PE)-precontracted aortic rings, PA induces vasodilation

PE-induced prolonged contraction in rat thoracic aorta rings was reduced by PA in a manner that is dependent on the concentration of the drug and on the presence of the endothelium. In both endothelium-intact and -denuded rings, the vasoconstriction elicited by 1 M phenylephrine was relaxed by the cumulative addition of doses of PA (2.77 × 10^−7^ to 2.04 × 10^−4^ M) in a concentration-dependent manner. In endothelium-denuded vascular rings, the concentration-response curve to PA was dramatically shifted to the right, with a statistically significant reduction in pEC_50_ after endothelium removal (pEC_50_ = 4.83 ± 0.14 vs pEC_50_ = 5.63 ± 0.15, *p*< 0.001, n = 5) (Figure 1B).

### Effects of L-name and indomethacin on praeruptorin-induced vasorelaxation

**Figure 2.**
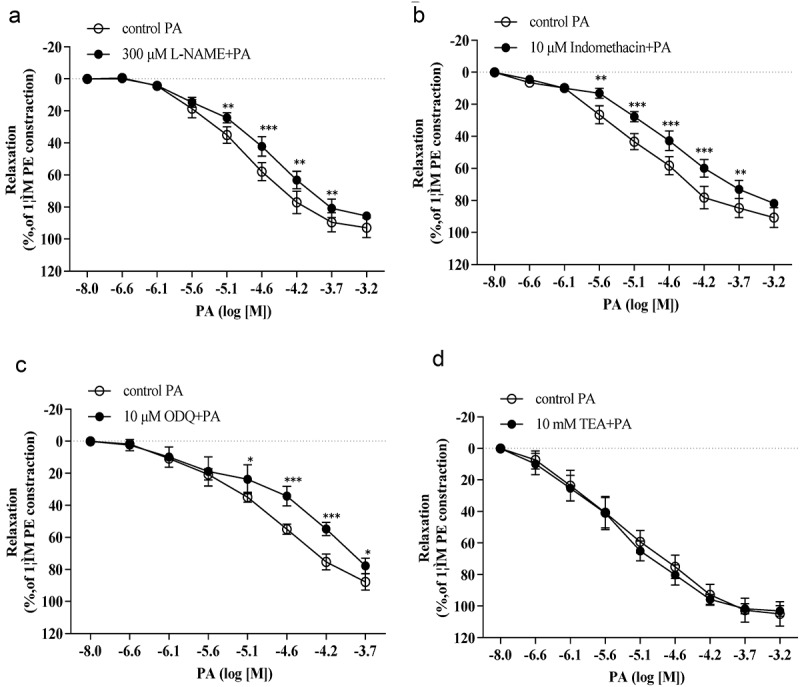
The relaxant effect of PA on PE (1 μM)-pre-contracted aortic rings in the presence or absence (control(PA), n = 4) of (A) 300 μM L-NAME, (B) 10 μM indomethacin, (C) 10 μM ODQ and (D) 10 μM on vasorelaxant effects of PA. Values are expressed as the mean ± SD. *p< 0.05, **p< 0.01, ***p< 0.001 vs the control group.

The PA-induced vasorelaxant response was reduced by both L-NAME (Figure 2A) and indomethacin (Figure 2B). After treatment with 300 μM L-NAME, the concentration-response curve to PA was significantly shifted to the right with a reduction in pEC_50_ (pEC_50_ = 4.57 ± 0.07 vs pEC_50_ = 4.86 ± 0.09, *p*< 0.01, *n* = 4). In the presence of 10 μM indomethacin, the concentration-response curve of PA was shifted to the right with a reduction in pEC_50_ (pEC_50_ = 4.58 ± 0.11 vs pEC_50_ = 4.99 ± 0.28, p< 0.05, *n*= 4), indicating that indomethacin suppressed the vasorelaxant effects of PA. Furthermore, there was no significant difference observed in the vasorelaxant effect of PA following L-NNA administration as compared to the control group (pEC_50_ = 4.90 ± 0.29 vs pEC_50_ = 5.02 ± 0.25, *p* > 0.05).

### Role of guanylyl cyclase inhibitor and K+ channel blocker in PA-induced vasorelaxation

We examined the vasorelaxant effect of PA after application of a guanylyl cyclase inhibitor (ODQ) and a KCa channel blocker (TEA). As shown in Figure 2C, ODQ inhibited PA-induced vasorelaxation. Further inhibition was observed after the application of 10 μM of ODQ, and the vasorelaxant effects of PA were significantly reduced (pEC_50_ = 4.40 ± 0.10 vs pEC_50_ = 4.89 ± 0.16, *n* = 4, *p* < 0.01). The data presented in Figure 2D demonstrate that the concentration-response curve for PA was not significantly changed after incubation with 10 mM of TEA.

### Effect of PA on calcium-induced vasoconstriction

**Figure 3.**
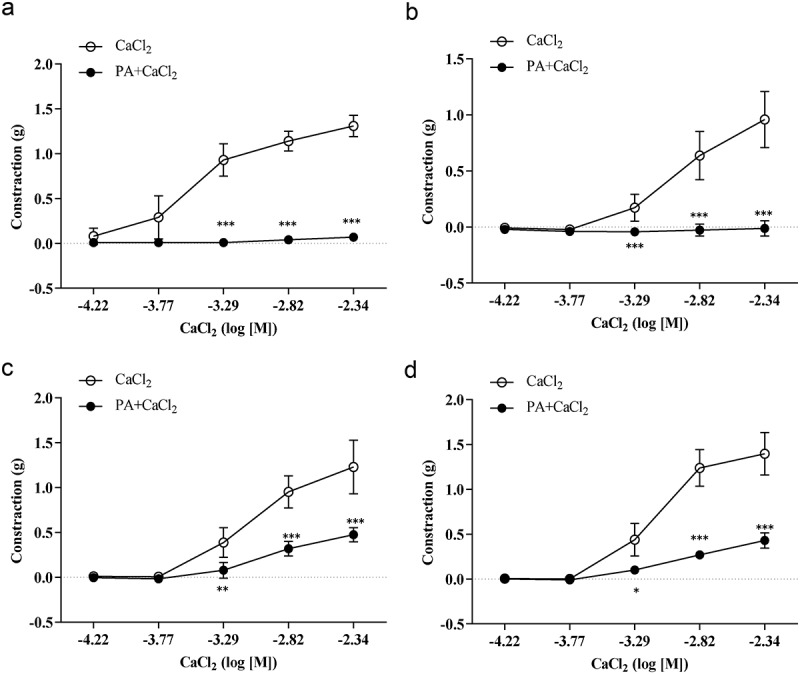
Effects of PA on calcium-induced vasoconstriction. Sustained contractile responses of 0.09 ± 0.02 g were induced by 60 mM KCl in intact thoracic aorta rings in K-H solution without Ca^2+^, and then CaCl_2_ (6.00 × 10^−5^ to 4.55 × 10^−3^ M) was cumulatively applied every 5 min. (A) Effect of PA on the cumulative contraction induced by influx of extracellular Ca^2+^ in intact thoracic aorta rings pre-contracted with KCl; (B) Effect of PA on contraction of endothelium-denuded aortic rings; (C) Effect of PA on the cumulative-contraction induced by influx of extracellular Ca^2+^ in intact thoracic aorta rings pre-contracted with PE; (D) Effect of PA on the cumulative-contraction induced by influx of extracellular Ca^2+^ in intact endothelium-denuded aorta rings pre-contracted with PE. Values are expressed as mean ± SD. *p < 0.05, **p < 0.01, ***p < 0.001 vs the Cacl_2_ alone group.

Sustained contractile responses of 0.09 ± 0.02 g were induced by 60 mM KCl in intact thoracic aorta rings in K-H solution without calcium in the presence or absence of PA (2.76 × 10^−^[5] M) when CaCl_2_ (6.00 × 10^−5^ to 4.55 × 10^−3^ M) was cumulatively applied every 5 min. As shown in Figure 3A, the maximum contractile response induced by CaCl_2_ was 1.31 ± 0.12 g; the calcium-induced maximum contraction was reduced by 94.66%, and the contractile response declined to 0.07 ± 0.02 g after incubation with PA (2.76 × 10^−^[5] M). In addition, the maximum contractile response induced by CaCl_2_ in endothelium-denuded thoracic aorta rings was 0.90 ± 0.25 g, and this response was reduced by 101.1% after PA (2.76 × 10^−^[5] M) incubation, where the contractile response decreased to −0.01 ± 0.007 g (Figure 3B). Sustained contractile responses of 0.10 ± 0.03 g were induced by 1 µM PE in intact thoracic aorta rings in K-H solution without calcium. When CaCl_2_ (6.00 × 10^−5^ to 4.55 × 10^−3^ M) was cumulatively applied every 5 min, the maximum contractile response induced by CaCl2 was 1.23 ± 0.29 g. After incubation with PA (2.76 × 10^−^[5] M), this maximum contractile response was decreased by 60.98%, and the contractile response changed to 0.07 ± 0.02 g (Figure 3C). The maximum contractile response induced by CaCl_2_ in endothelium-denuded thoracic aorta rings was 1.40 ± 0.24 g, which was reduced by 60.00% after incubation with PA (2.76 × 10^−5^ M) incubation and the contractile response decreased to 0.56 ± 0.18 g (Figure 3D).

## Discussion

Peripheral vascular resistance plays a fundamental role in the regulation of blood pressure. This resistance depends on vascular tone, which is the result of the balance between factors that influence vasoconstriction and vasodilation of smooth muscle cells. Excitatory stimuli increase calcium influx, which results in vascular smooth muscle contraction. However, vasodilation mainly relies on the endothelium. Once endothelial factors, such as NO, are stimulated and released, they reduce intracellular calcium levels and lead to relaxation of vascular smooth muscle [25], also called vasodilation. Here, we explored the relaxant effect of PA with regard to endothelial function.

First of all, we found that PA at concentrations as low as 2.77 × 10^−7^ M had a vasorelaxant effect in intact rat thoracic aorta rings; however, this effect was significantly reduced after removal of the vascular endothelium. This reduction supports the idea that the endothelium plays an important role in the process by which PA exerts a vasodilative effect.

The mechanisms underlying both vasoconstriction and vasodilation are complicated, and they involve membrane receptors and ion channels. The vascular endothelium plays an important role in the control of vascular tone [15], and it is a source of vasodilators, such as NO, prostacyclin, and other EDRFs [26]. Therefore, to clarify whether the levels of EDRFs, such as NO and Prostaglandin I2 (PGI2), influence the impact of PA on vasodilation, we incubated thoracic aorta artery rings with L-NAME, an NOS inhibitor, and indomethacin, an inhibitor of COX [27,28]. Concentration-response curves to PA were shifted to the right side with decreases of E_max_, which suggested that NO plays an important role in PA-induced vasodilation. With regard to PGI2, a COX metabolite, the relaxation induced by PA was significantly attenuated after treatment with indomethacin, which suggested that COX metabolites might be involved in the vasorelaxant response caused by PA. In short, EDRFs, in particular NO and PGI2, may play important roles in PA-induced vasodilation.

Multiple studies have demonstrated that NO-induced relaxation predominantly occurs through activation of soluble guanylyl cyclase in vascular smooth muscle, which leads to accumulation of cGMP and subsequent reduction of Ca^2+^ influx via cGMP-dependent protein kinase (PKG) activation [29,30]. Since we identified a role of NO in the vasodilative effect of PA, it was natural to further investigate the participation of the NO/cGMP pathway. We incubated preparations with ODQ, an inhibitor of NO-sensitive guanylyl cyclase. The results revealed that the vasorelaxant effects of PA were obviously decreased after ODQ administration, which implied that the NO/cGMP pathway may be involved in the relaxant response induced by PA.

Potassium channel activity may also be involved in vasodilatation via modulation of the cell membrane, inhibition of K^+^ outflow or blockage of voltage-dependent Ca^2+^ channels [31,32]. As mentioned above, calcium influx is one of the main factors responsible for vasoconstriction. If vasoconstriction is attenuated, conversely, vasodilation becomes relatively increased. Thus, we constructed an experiment from the perspective of vasoconstriction, mainly as mediated by calcium influx. The major finding of our work is that 2.76 × 10^−5^ M of PA inhibited CaCl_2_-induced vasoconstriction in both intact and denuded rat thoracic aorta rings, suggesting that Ca^2+^ may also contribute to the vasodilative effects of PA, in addition to the effects of the endothelium.

## Conclusion

PA appears to be an effective vasodilator in isolated rat thoracic aorta. The endothelium-dependent NO-cGMP pathway, the prostacyclin pathway and reduction of calcium influx may synergistically contribute to PA-mediated vasorelaxation.

## Data Availability

The datasets generated during the current study are available from the corresponding author upon reasonable request.
